# Clinical effectiveness of a modified muscle sparing posterior technique compared with a standard lateral approach in hip hemiarthroplasty for displaced intracapsular fractures (HemiSPAIRE): a multicenter, parallel-group, randomized controlled trial

**DOI:** 10.1136/bmjsit-2023-000251

**Published:** 2024-06-17

**Authors:** Susan Ball, Alex Aylward, Emma Cockcroft, Aisling Corr, Elizabeth Gordon, Alison Kerridge, Amy McAndrew, Sarah Morgan-Trimmer, Roy Powell, Anna Price, Shelley Rhodes, Andrew John Timperley, Jayden van Horik, Robert Wickins, John Charity

**Affiliations:** 1 NIHR Applied Research Collaboration South West Peninsula (PenARC), Department of Health and Community Sciences, University of Exeter Faculty of Health and Life Sciences, Exeter, UK; 2 NIHR Applied Research Collaboration South West Peninsula (PenARC), Patient Engagement Group, University of Exeter Faculty of Health and Life Sciences, Exeter, UK; 3 Department of Health and Community Sciences, University of Exeter Faculty of Health and Life Sciences, Exeter, UK; 4 Research & Development Department, Royal Devon University Healthcare NHS Foundation Trust, Exeter, UK; 5 Exeter Clinical Trials Unit, University of Exeter, Exeter, UK; 6 Research Design Service - South West, Royal Devon University Healthcare NHS Foundation Trust, Exeter, UK; 7 Exeter Hip Unit, Princess Elizabeth Orthopaedic Centre, Royal Devon University Healthcare NHS Foundation Trust, Exeter, UK; 8 Physiotherapy, Royal Devon University Healthcare NHS Foundation Trust, Exeter, UK

**Keywords:** Patient Outcome Assessment, Orthopedic Devices

## Abstract

**Objectives:**

Assess the effect of a modified muscle sparing posterior approach; SPAIRE (Save Piriformis and Internus, Repairing Externus), in hip hemiarthroplasty for displaced intracapsular fractures on postoperative mobility and function compared with a standard lateral approach.

**Design:**

Pragmatic, superiority, multicenter, parallel-group, randomized controlled trial (with internal pilot). Participants, ward staff, and research staff conducting postoperative assessments were blinded to allocation. A CTU allocated treatments centrally using computer-generated lists.

**Setting:**

Six hospitals in Southwest England, recruiting November 25, 2019–April 25, 2022.

**Participants:**

244 adults (≥60 years) requiring hip hemiarthroplasty (122 allocated to each approach). 90 and 85 participants allocated to SPAIRE and lateral, respectively, had primary outcome data within the prespecified data collection window.

**Interventions:**

Surgery using SPAIRE or standard lateral approach. Follow-up 3 days and 120 days postoperation.

**Main outcome measure:**

Oxford Hip Score (OHS), via telephone at 120 days. Secondary outcomes: function and mobility (3 days), pain (3 days, 120 days), discharge destination, length of hospital stay, complications and mortality (within 120 days), quality of life and place of residence (120 days).

**Results:**

Participants’ mean age was 84.6 years (SD 7.2); 168 (69%) were women. Primary outcome: little evidence of a difference in OHS at 120 days; adjusted mean difference (SPAIRE—lateral) −1.23 (95% CI −3.96 to 1.49, p=0.37). Secondary outcomes: indication of lower participant-reported pain at 3 days in SPAIRE arm; no differences between arms for remaining outcomes.

**Conclusions:**

Participants’ mobility and function are similar in the short term (3 days) and longer term (120 days), whether receiving the SPAIRE or lateral approach. Neither approach confers benefit over the other in terms of length of hospital stay, return to prefracture residence, survival within 120 days, or quality of life at 120 days. Participants receiving SPAIRE approach may experience less pain in the early postoperative period. Modifying the posterior approach in hip hemiarthroplasty to the SPAIRE approach gives equivalent patient outcomes to the lateral approach within 120 days.

**Trial registration number:**

NCT04095611.

**WHAT IS ALREADY KNOWN ON THIS TOPIC**

- The National Institute for Health and Care Excellence recommends hemiarthroplasty as the procedure of choice for the treatment of displaced intracapsular fractures when patients are not eligible for total hip replacement and recommends that hemiarthroplasties are carried out using a lateral approach rather than a conventional posterior approach.
- Literature suggests a reduced dislocation rate for the lateral approach, compared with the conventional posterior approach, but with the disadvantage that the extensive division of tendon attachments results in reduced levels of function postoperatively.
- A modified technique using a posterior approach; SPAIRE (Save Piriformis and Internus, Repairing Externus), which is muscle sparing and has enhanced capsule repair, aims to provide sufficient stability to enable patients to mobilize full weight bearing, without the specific restrictions currently included in routine postoperative posterior approach protocols.

**WHAT THIS STUDY ADDS**

- HemiSPAIRE showed no difference between the SPAIRE and lateral approach to hip hemiarthroplasty in terms of participants’ mobility and function, as assessed by the patient-reported Oxford Hip Score at 120 days postoperation.
- Participants’ mobility and function are similar in the short term (3 days postoperation), regardless of whether they receive the SPAIRE or lateral surgical approach to hip hemiarthroplasty and neither surgical approach confers benefit over the other in terms of length of acute or total hospital stay, return to prefracture place of residence, survival within 120 days postoperation, or quality of life at 120 days postoperation.
- Participants receiving the SPAIRE approach to hip hemiarthroplasty may experience less pain in the early postoperative recovery period, compared with those receiving the lateral approach.

**HOW THIS STUDY MIGHT AFFECT RESEARCH, PRACTICE OR POLICY**

- Modification of the posterior approach in hip hemiarthroplasty following the SPAIRE technique can be carried out with equivalent patient outcomes to the lateral approach within 120 days post-operation.
- Surgeons involved in the care of hip fracture patients requiring hemiarthroplasty who routinely use the posterior approach to the hip may find the SPAIRE technique of interest, given the familiarity with the relevant anatomy and potential for reduced pain in the early postoperative period.
- Current NICE guidelines recommend surgeons consider the lateral approach in hip hemiarthroplasty in favor of the conventional posterior approach. Findings from this and other studies show the SPAIRE modification of the posterior approach is safe and equivalent to the standard lateral approach in terms of the outcomes measured. NICE may wish to consider including the SPAIRE modification of the posterior approach as equivalent to the standard lateral approach in its recommendation for surgery.
- The challenges that the population of England experienced in accessing community rehabilitation during the COVID-19 pandemic are likely to have impacted patient outcome at 120 days postoperation. Findings from our qualitative work reflected this impact of access to physiotherapy on return to function and should be considered in future work.

## Introduction

Out of approximately 65,000 people with hip fracture treated annually in England, Wales and Northern Ireland, over 20,000 hip hemiarthroplasties (replacing the fractured head on the upper end of the femur) are performed.^1^ People with hip fracture endure debilitating loss of function and recovery is challenging.^2^ The average length of stay nationally for hip fracture admissions is over 21 days, representing over 4000 hospital beds occupied at any one time.^3^

The National Institute for Health and Care Excellence (NICE) recommends hemiarthroplasty as the procedure of choice for the treatment of displaced intracapsular fractures when patients are not eligible for total hip replacement and recommends that hemiarthroplasties are carried out using a lateral approach rather than a conventional posterior approach.^4^ For adequate exposure, the lateral approach requires division and repair of ≥50% tendon attachments of the gluteus medius and minimus muscles on to the greater trochanter—muscles essential for walking. Literature suggests a reduced dislocation rate for the lateral approach, but with the disadvantage that extensive division of tendon attachments results in reduced levels of function postoperatively. Recent evidence from a cohort of 20,908 patients from the Norwegian Hip Fracture Register reported better patient-related outcomes (pain, patient satisfaction, health-related quality of life) with a conventional posterior approach compared with the lateral approach.^5^ Results from a cohort of 25,678 patients in the Swedish Hip Arthroplasty Registry reported higher dislocation rates in patients receiving the posterior approach, where 366/13,769 (2.7%) patients operated on with the lateral approach dislocated, compared with 850/11,834 (7.2%) of those with the posterior approach.^6^ To address the issue of instability leading to dislocation, modifications to surgical approaches have been attempted using minimally invasive and muscle sparing techniques. Han *et al* described a modified posterior approach for use in patients with neurological disorders requiring hip hemiarthroplasty, where the piriformis, gemellus superior, obturator internus and part of quadratus femoris muscles were left intact, which combined with a standard capsule repair led to a reduced incidence of dislocation.^7^ In 2016, the Hip Unit at the Royal Devon University Healthcare National Health Service Foundation Trust developed a modified technique using a posterior approach, applicable to all patients, named SPAIRE; Save Piriformis and Internus, Repairing Externus. Piriformis and Internus muscles have been shown to act as the main extensor and abductor of the flexed hip which is important for movements such as rising from a chair or climbing stairs.^8^ This contrasts with the standard lateral approach, where a significant proportion of the gluteal muscle insertions is divided, potentially impacting hip function. The combination of a muscle sparing approach with enhanced capsule repair in the SPAIRE technique aims to provide sufficient stability to enable patients to mobilize full weight bearing, without the specific restrictions currently included in routine postoperative posterior approach protocols.

The primary trial objective was to test whether the SPAIRE technique improved postoperative function and mobility, in terms of the Oxford Hip Score (OHS), at 120 days after surgery in adults aged ≥60 years, with a displaced intracapsular hip fracture requiring hemiarthroplasty, compared with the standard lateral approach. Secondary objectives were to (1) test whether the SPAIRE technique resulted in improved early function, mobility and pain, pain and quality of life at 120 days, length of hospital stay and complication rates and mortality up to 120 days following surgery compared with the standard lateral approach, through collecting secondary outcome measures; (2) investigate how participants experienced their recovery period after surgery, and investigate mechanisms of recovery, including experience of postoperative pain and engagement in physiotherapy, by conducting a qualitative study with a subsample of participants in each trial arm; and (3) work with patients and carers with relevant lived experience to ensure the conduct and outputs of the study are relevant and useful to patients who receive hemiarthroplasty surgery.

## Methods

### Trial design

HemiSPAIRE was a pragmatic, superiority, parallel-group, randomized controlled trial (RCT) (with internal pilot), with blinded assessment, comparing patient outcomes under the SPAIRE and standard lateral approaches to hip hemiarthroplasty for displaced intracapsular fractures. The trial included an embedded qualitative study. The protocol and statistical analysis plan (SAP) are published.^9 10^ Conduct and reporting followed Consolidated Standards of Reporting Trials guidelines.^11^ All randomized participants provided informed consent. An independent trial steering committee (TSC) reviewed progress against prespecified stop-go criteria^9^ and throughout the trial.

### Participants

Participants attending one of six hospitals in South West England with a displaced intracapsular fracture requiring hip hemiarthroplasty were recruited. If potentially eligible, the study was discussed with the patient and/or their carer(s). Potential participants were identified by orthopedic surgeons involved in the study, who admitted patients under their care. Identified patients interested in participating were invited to read the participant information sheet and, if interested, to provide informed consent. If a patient lacked capacity and was unable to consent, potential participation was discussed with a healthcare professional who was involved in the patient’s care but not part of the research team, and if deemed appropriate a declaration was signed as well as seeking the opinion of a person whose relationship to the patient made them suitable to act as his/her legal representative. Participants who lacked the mental capacity to consent and did not seem in agreement with any part of the study, even if agreement had been given by another, were not included.

Patients were eligible if they were aged ≥60 years, presented with an intracapsular hip fracture requiring hip hemiarthroplasty, and were resident in South West England. Exclusion criteria were being immobile (unable to walk) before hip fracture; not expected to live until postoperative day 120 due to chronic illness and receiving surgery for palliative care; or use of femoral stems not of a proven stem design, in line with NICE clinical guideline on hip fracture management.^4^ A sizeable proportion of this population suffers dementia and/or temporary delirium and was not excluded. A systematic review of observational studies found that 19% of people with hip fracture meet formal diagnostic criteria for dementia, and 42% are cognitively impaired.^12^ Cognitive ability was not part of the HemiSPAIRE eligibility criteria.

### Randomization and blinding

Randomization was undertaken as late as practically possible. There was no special preparation required in theater, and no difference in equipment required for either surgical technique. Participants were individually randomized to receive the SPAIRE or lateral procedure in a 1:1 ratio. Concealed allocation was determined by the UKCRC registered Exeter Clinical Trials Unit (CTU) using a validated password-protected web-based system, based on random permuted blocks of varying size (2, 4 or 6). Stratification variables were hospital site and cognition level (impaired vs non-impaired), from information gathered by the research nurse in the participant’s records. The surgeon was informed of allocation by the CTU via email through nhs.net mail. The trial chief investigator and the principal investigator at the hospital site were copied in. Participants, ward staff, all research staff involved in assessments, and the trial statistician, were blinded to treatment allocation. Surgeons and operative teams were unblinded. There is no difference between the SPAIRE and lateral approach techniques in the following: preparation for surgery, patient positioning, skin incision, surgical time taken, application of surgical dressing or postoperative care. For medicolegal reasons, the surgical approach used was specified in the operation notes. A cover sheet was attached to the front of the printed operation notes stating that the patient is a trial participant and reminding the research team to avoid inadvertent unblinding to treatment allocation during postoperative assessments. Provision of information on the study, consenting, randomization and postoperative assessments were carried out by research teams at each site.

### Procedures

The SPAIRE technique via the posterior approach to the hip involved a modified muscle sparing posterior approach where insertions of piriformis, superior gemellus, obturator internus and inferior gemellus were spared with division of only obturator externus and part of quadratus femoris. The single divided tendon and posterior capsule were subsequently repaired with a transosseous repair to their initial position prior to closure. The insertions of the abductor muscles were left intact throughout the procedure. Further details of the SPAIRE technique are published elsewhere.^13^ In the lateral approach, the operation was performed in accordance with criteria set by the study, to minimize issues of standardization with this approach. This means that the gluteus medius and minimus insertions onto the greater trochanter were partially divided anteriorly, leaving the posterior part of their insertions intact. The anterior capsule was divided and subsequently repaired prior to closure, followed by repair of the detached portion of the gluteal muscles.

### Outcomes

The primary outcome was function and mobility, reported by the participant (or by proxy by a family member or carer if the participant was unable to answer the questions), measured using the OHS (range 0–48; 48 is the best score),^14 15^ at 120 days postoperation. The 120-day follow-up was chosen for the primary outcome because it is the time of final review offered to all hip fracture patients by the National Hip Fracture Database. Secondary outcomes included level of function and mobility at 3 days postoperation (and 120 days if possible) using the De Morton Mobility Index (DEMMI) (range 0–100; 100 is best score),^16^ early (3 days) mobility using the Cumulated Ambulation Score (CAS) (range 0–18; 18 is best score),^17^ level of pain using a Numeric Pain Rating Scale (NPRS) from 0 (no pain) to 10 (worst possible pain) at 3 and 120 days, health-related quality of life (European Quality of Life 5 Dimensions 5 Level, EQ-5D-5L) (range <0 (health state worse than death) to 1 (full health), with 0 being value of a heath state equivalent to dead)^18 19^ at 120 days, acute and total length of hospital stay, hip-related complications and mortality within 120 days of operation, acute discharge destination, and place of residence at 120 days. Participants were assessed face to face at 3 days and via telephone at 120 days, by research nurses.

### Sample size

The original primary outcome was the DEMMI, and the trial was powered to detect a difference of 6 points^16^ between trial arms, at 120 days (assuming an SD of 11.9), with 90% power, at the 5% level of significance, allowing for 25% loss to follow-up, giving a recruitment target of 224 participants (112 in each arm). The primary outcome was changed to the OHS (non-substantial amendment approved by the sponsor June 17, 2020 and acknowledged by the Health Research Authority (HRA)). This was because the DEMMI must be assessed in person with the participant, requiring either an outpatient appointment or home visit, which were not possible during periods of COVID-19 restrictions. The OHS can be collected remotely, via the telephone. The recruitment target was updated, based on a minimal clinically important difference for OHS of 5 points^20^ and an SD of 10 (ie, an effect size of 0.5), with 90% power, at the 5% level of significance, requiring 85 participants per trial arm, that is, a total of 170. Allowing for 25% loss to follow-up, the recruitment target was 228. Review of follow-up rates by the study team during the trial indicated that drop-out rates were higher than originally anticipated and as such the recruitment target was updated again (non-substantial amendment approved by the sponsor December 2, 2021 and acknowledged by the HRA), to allow for a 30% loss to follow-up, requiring a total of 244 participants (122 in each arm).

### Statistical analysis

Statistical analyses are detailed in the published SAP.^10^ The SAP was prespecified and approved prior to final data collection. To aid presentation of trial findings to the trial team, the trial statistician was unblinded at the point of receiving the final trial data, which was after all data queries had been resolved and the trial database had been locked. Analyses were completed using Stata version V.17.0 (StataCorp).

Comparisons of outcomes between trial arms used the all-randomized population, under the intention-to-treat (ITT) principle with participants analyzed according to the trial arm they were randomized to.

The main analyses used a survivor average causal effect (SACE) approach, which allowed estimation of the effect of surgical approach on outcomes in the population of people who would have survived regardless of what surgical approach they received.^21^ Further details of this method are in the published SAP.^10^ The main analyses were based on the complete case data. The adjusted analyses are the main analyses. Analyses based on multiple imputed datasets were conducted as additional analyses. Multiple imputation was used to impute missing data, under the assumption that data were missing at random according to Rubin’s rules,^22^ using the chained equations approach and predictive mean matching.^23^ 50 imputed datasets were generated. Variables used to impute missing data included all outcomes at all follow-up time points, trial arm status, stratification variables, and variables included as adjustment factors in the regression models fitted to outcomes. While all participants were included in the imputation process, no outcomes were imputed for participants who died before the outcome could have been assessed. Further additional analyses were conducted. First, using the trial data (ie, not using multiply imputed data): (1) Analyses using linear (for continuous outcomes) and logistic (for binary outcomes) regression, among those participants who survived—a survivors analysis. The population of interest comprised participants who survived under the surgical approach they received. (2) Analyses using linear and logistic regression, setting those participants who died before the outcome could be assessed to the worst possible score (for continuous outcomes OHS, DEMMI, CAS, NPRS), the score equivalent to being dead (for EQ-5D-5L), the worst score observed among all participants (for lengths of stay), or the worst category (for binary outcomes discharged to prefracture residence, and living at prefracture place of residence at 120 days)—a composite approach. The population of interest comprised all participants regardless of whether they survived or not. (3) Analyses including any outcomes collected outside the prespecified data collection window (ie, outside days 110–130), using a SACE approach. (4) Analyses of all outcomes that could be completed by proxy, excluding those participants for whom data collection was by proxy, using a SACE approach. Second, the following analyses were repeated, based on multiple imputed datasets: (1) main analyses using a SACE approach; (2) survivors analyses; and (3) composite analyses.

### Patient and public involvement

Patient and public involvement (PPI) helped shape the trial and inform the primary outcome and premise of the work. During trial development, meetings were held with patients to discuss the importance of the research, recruitment and acceptability of outcome measures. It was clear that mobility was the highest priority. Patient/carer coapplicant (AA) was involved from conception of the trial idea and as a trial management group member, AA worked closely with the trial team, including the PPI facilitator (EC), ensuring a patient focus throughout. PPI was integrated throughout the trial from design to dissemination. NIHR INVOLVE guidance for good practice for effective involvement was followed, including payment and reimbursement of expenses for patient advisors. An advisory group of two people with experience of caring for a family member after a hip fracture met on three occasions, with additional correspondence via email. This group informed the production of study materials, gave feedback on the qualitative interview schedule and was involved in the interpretation of findings.

### Qualitative study

The embedded qualitative study examined participants’ experiences of their postoperative recovery period and compared findings between trial arms. 18 semistructured telephone interviews were conducted by one researcher (EC). A subsample of trial participants (8 in SPAIRE arm and 10 in lateral arm) were recruited from 3 sites. Participants were interviewed around 130 days after surgery. Interviewees were asked about impact of surgery and experience of recovery during the postoperative period.

Interviews were transcribed verbatim and uploaded to NVivo. Data were analyzed using adapted thematic analysis, employing a combined deductive-inductive approach to explore known factors that would be experienced during recovery but also allow for unexpected findings.^24^ Four interviews were double-coded (SM-T, EC) and SM-T and EC met periodically during the analysis to develop and refine codes and themes. Findings were then compared between trial arms by EC and SM-T. Researchers were blinded to the trial arm of interviewees until analyses were complete.

## Results

**Figure 1:**
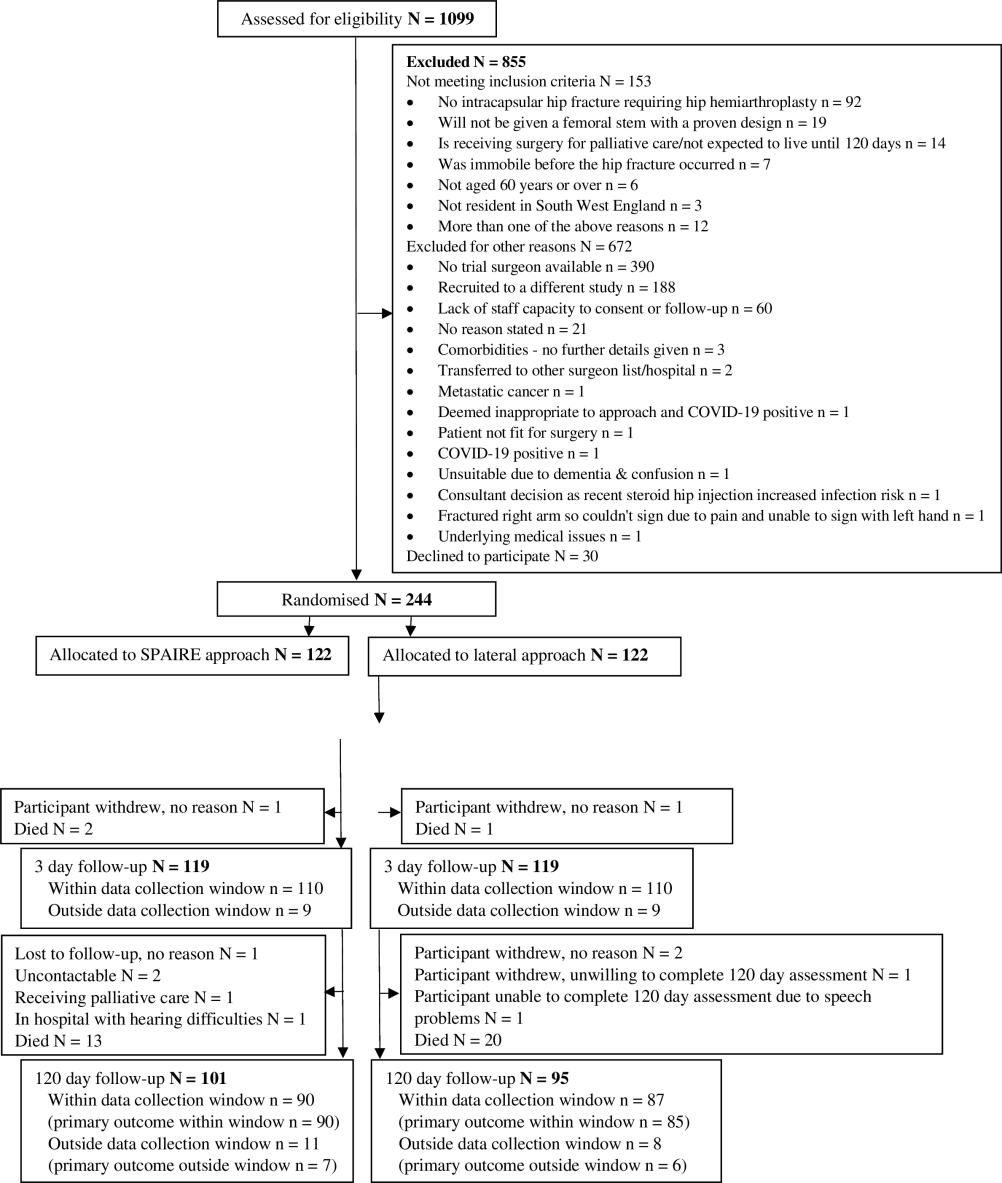
CONSORT trial profile. CONSORT, Consolidated Standards of Reporting Trials; SPAIRE, Save Piriformis and Internus, Repairing Externus.

Between November 25, 2019 and April 25, 2022, 1099 patients were assessed for eligibility and 244 were randomly assigned to 1 of the 2 trial arms: 122 to SPAIRE and 122 to lateral (figure 1). Among 153 patients who did not meet the inclusion criteria, most did not have an intracapsular hip fracture requiring hemiarthroplasty (n=92, 60%). Of the 702 eligible non-participants, the most common reasons that patients were not randomized were no trial surgeon being available (n=390, 56%) or the patient being recruited to a different study (n=188, 27%).

Trial participants were similar to eligible non-participants with respect to gender and age (online supplemental table S1). Baseline (prefracture) characteristics were similar between the two trial arms. The mean age of participants was 84.6 years (SD 7.2) and 168 (69%) were women (table 1).

10.1136/bmjsit-2023-000251.supp2 Supplementary data

**Table 1:** Participant baseline characteristics by trial arm status and overall

Baseline characteristic	SPAIRE approach (N=122)	Lateral approach (N=122)	Overall (N=244)
Sex			
Female, n (%)	85 (69.7)	83 (68.0)	168 (68.9)
Male, n (%)	37 (30.3)	39 (32.0)	76 (31.1)
Age in years, mean (SD)	84.5 (7.4)	84.7 (7.0)	84.6 (7.2)
Ethnicity			
White British, n (%)	122 (100)	121 (99.2)	243 (99.6)
American, n (%)		1 (0.8)	1 (0.4)
Cognition level			
Impaired, n (%)	36 (29.5)	35 (28.7)	71 (29.1)
Not impaired, n (%)	86 (70.5)	87 (71.3)	173 (70.9)
Place of residence			
Own home, n (%)	98 (80.3)	96 (78.7)	194 (79.5)
Residential/supported living, n (%)	18 (14.8)	23 (18.9)	41 (16.8)
Nursing home, n (%)	6 (4.9)	3 (2.5)	9 (3.7)
ASA score			
1, n (%)	1 (0.8)		1 (0.4)
2, n (%)	34 (27.9)	32 (26.4)	66 (27.2)
3, n (%)	73 (59.8)	73 (60.3)	146 (60.1)
4+, n (%)	14 (11.5)	16 (13.2)	30 (12.3)
OHS, mean (SD)	38.7 (7.8)*	37.5 (8.1)*	38.1 (7.9)
EQ-5D-5L			
Index value, mean (SD)	0.78 (0.20)†	0.76 (0.23)‡	0.77 (0.22)
VAS, mean (SD)	68.45 (20.65)§	66.29 (22.22)¶	67.37 (21.41)
Recruitment site			
A, n (%)	59 (48.4)	57 (46.7)	116 (47.5)
B, n (%)	33 (27.0)	36 (29.5)	69 (28.3)
C, n (%)	11 (9.0)	10 (8.2)	21 (8.6)
D, n (%)	7 (5.7)	9 (7.4)	16 (6.6)
E, n (%)	8 (6.6)	6 (4.9)	14 (5.7)
F, n (%)	4 (3.3)	4 (3.3)	8 (3.3)

*N=108.

†N=99.

‡N=96.

§N=92.

¶N=91.

ASA, American Society of Anesthesiologists; EQ-5D-5L, European Quality of Life 5 Dimensions 5 Level; OHS, Oxford Hip Score; SPAIRE, Save Piriformis and Internus, Repairing Externus; VAS, Visual Analogue Scale.

Baseline characteristics of participants for whom the primary outcome was collected within the prespecified data collection window were similar between trial arms. These participants had similar characteristics to those for whom the primary outcome was collected outside the data collection window, both within trial arm and overall. Among those participants who did not survive to 120 days, a higher proportion were cognitively impaired and were living in residential/supported living or a nursing home, compared with those who survived (online supplemental table S2).

In the lateral arm, one participant died before surgery and five participants either did not have their operation performed by a trial surgeon (n=2), had a hemiarthroplasty performed under an approach that was not the standard lateral (standard posterior n=1), or had an operation that was not a hemiarthroplasty (n=2). These five participants were analyzed according to the approach they were randomized to, under the ITT principle. Five participants withdrew and 21 died during the follow-up period. All 122 participants in the SPAIRE arm received the SPAIRE approach, performed by a trial surgeon. One participant withdrew, 5 were lost to follow-up and 15 participants died during the follow-up period.

Following satisfaction of the prespecified stop-go criteria,^9^ the TSC approved continuation of the trial beyond the pilot phase. The trial ended when the final participant follow-up was completed on August 19, 2022.

### Primary outcome measure

The primary outcome at 120 days was collected on 188 of the 244 (77%) randomized participants. Of these, 175 participants had data collected within the data collection window and were included in the main analysis, which showed little evidence of an effect of surgical approach on the OHS. The mean (SD) OHS at 120 days was 32.2 (9.3) in the SPAIRE arm and 33.7 (8.3) in the lateral arm. The adjusted mean difference (SPAIRE—lateral) was −1.23 (95% CI −3.96 to 1.49, p=0.37). The effect size (adjusted mean difference/pooled SD) was −0.14. Additional analyses of the primary outcome showed similar results (table 2).

**Table 2:** Difference in OHS between SPAIRE and lateral trial arms at 120 days after surgery

Analysis approach*	SPAIRE		Lateral		Unadjusted	Adjusted		
	N†	Mean (SD)†	N†	Mean (SD)†	Mean difference	Mean difference	95% CI	P value
Main analysis: SACE	90	32.24 (9.27)	85	33.66 (8.28)	−1.26	−1.23	−3.96 to 1.49	0.37
Additional analyses:								
Survivors	90	32.24 (9.27)	85	33.66 (8.28)	−1.42	−1.44	−4.07 to 1.19	0.28
Composite	105	27.64 (14.22)	107	26.74 (15.53)	0.90	0.19	−3.53 to 3.91	0.92
SACE, including OHS outside 110–130 days	97	32.31 (9.12)	91	33.37 (8.26)	−0.87	−0.98	−3.58 to 1.62	0.46
SACE, excluding OHS collected by proxy	70	33.20 (9.43)	60	34.68 (7.51)	−1.72	−1.51	−4.61 to 1.59	0.34
SACE, multiply imputed data sets (N_SPAIRE_=100; N_lateral_=94)‡	90	32.24 (9.27)	85	33.66 (8.28)	−1.56	−1.60	−4.30 to 1.10	0.25
Survivors, multiply imputed data sets (N_SPAIRE_=100; N_lateral_=94)‡	90	32.24 (9.27)	85	33.66 (8.28)	−1.65	−1.77	−4.40 to 0.85	0.18
Composite, multiply imputed data sets (N_SPAIRE_=105; N_lateral_=109)‡	105	27.64 (14.22)	107	26.74 (15.53)	1.23	0.25	−3.33 to 3.83	0.89

*All analyses are on the intention-to-treat basis, adjusted for site, cognition level and prefracture characteristics age, gender, place of residence, ASA score. Main analysis is an SACE analysis of complete-case data. Survivors analysis is a linear model fitted to OHS of participants who were alive at 120-day follow-up. Composite analysis is a linear model fitted to OHS, having assigned those participants who died before the 120-day follow-up an OHS of zero. SACE, including OHS outside 110–130 days, is the same as the main analysis but including OHS collected outside the prespecified data collection window. SACE, excluding OHS collected by proxy, is the same as the main analysis but excluding OHS collected by proxy. SACE, multiply imputed data sets, is the main analysis with 50 imputed data sets derived with multiple imputation by chained equations and results combined using Rubin’s rules. While data on all participants are used in the multiple imputation process, outcome data are not imputed for participants who died before the 120-day follow-up. Survivors, multiply imputed data sets analysis, is the survivors analysis with 50 imputed data sets as described above. Composite, multiply imputed data sets analysis, is the composite analysis with 50 imputed data sets as described above.

†Sample sizes and summary statistics from original trial data.

‡Sample sizes after multiple imputation.

ASA, American Society of Anesthesiologists; OHS, Oxford Hip Score; SACE, survivor average causal effect; SPAIRE, Save Piriformis and Internus, Repairing Externus.

### Secondary outcomes

Main analyses of the secondary outcomes showed little evidence of a difference between trial arms in terms of pain and quality of life at 120 days, length of acute and total hospital stay, and return to pre-fracture place of residence at acute discharge and at 120 days (table 3). Additional analyses of secondary outcomes showed similar results (online supplemental tables S3–S5), with the exception of the NPRS, for which there was some evidence that participants in the SPAIRE arm reported less pain at 120 days than those in the lateral arm when using (1) the main analytical (SACE) approach based on complete-case data and including outcomes collected outside the prespecified data collection window (adjusted mean difference (SPAIRE—lateral) −1.00, 95% CI −1.91 to −0.10, p=0.03) and (2) the composite approach based on multiple imputed datasets (adjusted mean difference (SPAIRE—lateral) −1.02, 95% CI −2.03 to −0.01, p=0.048). All analyses of the NPRS at 3 days showed some evidence of an effect of surgical approach on this outcome, with participants in the SPAIRE arm reporting lower pain than those in the lateral arm (table 4). Analgesia use at 3 days was reported in the patient notes of 119 participants in each trial arm and showed similar numbers of participants taking medications within the last 24 hours (116 and 109 participants in the SPAIRE and lateral arm, respectively). At 120 days, 45 of 101 participants who were followed up in the SPAIRE arm, and 43 of 95 participants who were followed up in the lateral arm, had taken some form of analgesic in the last 24 hours.

**Table 3:** Difference in secondary outcomes between SPAIRE and lateral trial arms using a survivor average causal effect approach

Outcome	SPAIRE		Lateral		Statistic	Unadjusted	Adjusted*		
	N	Mean (SD)/n (%)	N	Mean (SD)/n (%)		Estimate	Estimate	95% CI	P value
NPRS: 120 days	74	1.70 (2.40)	69	2.61 (3.09)	Mean difference	−0.87	−0.92	−1.88 to 0.04	0.06
EQ-5D-5L: 120 days									
Index value	83	0.76 (0.24)	82	0.73 (0.22)	Mean difference	0.02	0.02	−0.05 to 0.09	0.55
VAS	77	66.65 (19.65)	74	69.53 (19.60)	Mean difference	−3.14	−2.97	−9.48 to 3.53	0.37
Length of stay (acute)	115	14.05 (16.11)	116	12.66 (7.45)	Mean difference	1.73	1.55	−1.65 to 4.75	0.34
Length of stay (total)	114	19.41 (20.95)	115	19.28 (14.95)	Mean difference	1.18	1.10	−3.70 to 5.90	0.65
Discharged from acute stay to prefracture place of residence	115	73 (63.48)	116	63 (54.31)	OR	1.40	1.43	0.79 to 2.60	0.24
Living in same place of residence at 120 days as prefracture	90	76 (84.44)	87	73 (83.91)	OR	1.15	1.13	0.47 to 2.73	0.79

All analyses use a survivor average causal effect approach, based on complete case data, under the intention-to-treat principle. Analyses of length of stay (acute) and discharged from acute stay to prefracture residence exclude those who died during acute stay. Analysis of length of stay (total) excludes those who died during acute or total stay.

*For NPRS, EQ-5D-5L, lengths of stay and discharged from acute stay to same place of residence as prefracture: analyses adjusted for site, cognition level and prefracture characteristics age, gender, place of residence, ASA score. For living in same place of residence at 120 days as prefracture: adjusted for site and cognition level.

ASA, American Society of Anesthesiologists; EQ-5D-5L, European Quality of Life 5 Dimensions 5 Level; NPRS, Numeric Pain Rating Scale; SPAIRE, Save Piriformis and Internus, Repairing Externus; VAS, visual analogue scale.

**Table 4:** Additional analyses comparing the Numeric Pain Rating Scale between SPAIRE and lateral trial arms

Analysis approach*	SPAIRE		Lateral		Unadjusted	Adjusted		
	N†	Mean (SD)†	N†	Mean (SD)†	Mean difference	Mean difference	95% CI	P value
3 days								
Survivors, complete case	91	4.29 (2.71)	81	5.23 (2.92)	−0.95	−0.99	−1.84 to −0.13	0.02
Composite, complete case	93	4.41 (2.81)	83	5.35 (2.98)	−0.94	−0.90	−1.78 to −0.03	0.04
Survivors, multiply imputed data sets (N_SPAIRE_=115; N_lateral_=116)‡	91	4.29 (2.71)	81	5.23 (2.92)	−0.83	−0.88	−1.74 to −0.03	0.04
Composite, multiply imputed data sets (N_SPAIRE_=112; N_lateral_=112)‡	93	4.41 (2.81)	83	5.35 (2.98)	−0.85	−0.90	−1.73 to −0.07	0.04
120 days								
Survivors, complete case	74	1.70 (2.40)	69	2.61 (3.09)	−0.91	−0.92	−1.86 to 0.02	0.054
Composite, complete case	89	3.10 (3.81)	91	4.40 (4.17)	−1.29	−0.99	−2.07 to 0.10	0.07
SACE, complete case, including NPRS outside 110–130 days	80	1.70 (2.44)	75	2.73 (3.12)	−0.99	−1.00	−1.91 to −0.10	0.03
SACE, multiply imputed data sets (N_SPAIRE_=101; N_lateral_=94)‡	74	1.70 (2.40)	69	2.61 (3.09)	−0.78	−0.80	−1.68 to 0.08	0.08
Survivors, multiply imputed data sets (N_SPAIRE_=101; N_lateral_=94)‡	74	1.70 (2.40)	69	2.61 (3.09)	−0.80	−0.77	−1.65 to 0.11	0.09
Composite, multiply imputed data sets (N_SPAIRE_=105; N_lateral_=109)‡	89	3.10 (3.81)	91	4.40 (4.17)	−1.22	−1.02	−2.03 to −0.01	0.048

*All analyses are on the intention-to-treat basis, adjusted for site, cognition level and prefracture characteristics age, gender, place of residence, ASA score. Survivors analysis is a linear model fitted to NPRS of participants who were alive at 120-day follow-up. Composite analysis is a linear model fitted to NPRS, having assigned those participants who died before the 120-day follow-up an NPRS of 10. SACE, including NPRS outside 110–130 days, is the same as the main analysis but including NPRS collected outside the prespecified data collection window. SACE, multiply imputed data sets, is the main analysis with 50 imputed data sets derived with multiple imputation by chained equations and results combined using Rubin’s rules. While data on all participants are used in the multiple imputation process, outcome data are not imputed for participants who died before the 120-day follow-up. Survivors, multiply imputed data sets, is the survivors analysis with 50 imputed data sets as described above. Composite, multiply imputed data sets, is the composite analysis with 50 imputed data sets as described above.

†Sample sizes and summary statistics from original trial data.

‡Sample sizes after multiple imputation.

ASA, American Society of Anesthesiologists; NPRS, Numeric Pain Rating Scale; SACE, survivor average causal effect; SPAIRE, Save Piriformis and Internus, Repairing Externus.

Analysis of time to death based on the ITT population showed little evidence of differential survival between trial arms (adjusted HR 0.68, 95% CI 0.35 to 1.35, p=0.27). Results were similar based on per protocol data (ie, including those participants who had the surgical approach they were randomized to, performed by a trial surgeon). The per protocol dataset included 33 deaths (14 SPAIRE, 19 lateral). There was little evidence of differential survival between trial arms (adjusted HR 0.72, 95% CI 0.36 to 1.45, p=0.36).

Hip-related complications deemed serious adverse events (SAEs) during the trial included three periprosthetic fractures (all in the lateral arm), two dislocations (one per arm), one infection (SPAIRE arm) and one participant had nerve palsy (lateral arm). In total, 42 (34.4%) participants in the SPAIRE arm and 46 (37.7%) in the lateral arm had at least one SAE, including death. There were 280 AEs: 127 in the SPAIRE arm (among 63 (51.6%) participants) and 153 in the lateral arm (among 59 (48.4%) participants).

### Qualitative study findings

Interviewees’ experiences of recovery were similar between trial arms. Four themes were developed in the analysis:

#### Theme 1: steady but limited recovery

Interviewees generally experienced recovery as gradual improvement and summarized their recovery in positive terms. Most were still affected by incomplete aspects of recovery such as ongoing pain or unsteadiness and still had indoor and outdoor mobility difficulties.

Many interviewees were relying on support from family and were using mobility aids such as walking frames or adaptations in their homes. Some experienced anxiety about falling, due to the accident resulting in hip fracture but also pre-existing health conditions or living alone.

Most interviewees felt their quality of life was affected by reduced mobility or being ‘slower’ than before. This was usually attributed to either their hip fracture or age. A few interviewees had been told recovery overall could take 6–12 months and viewed their lack of mobility in this context, though others were frustrated with the speed of their recovery.

#### Theme 2: characterization of pain and low mental well-being as not severe

Interviewees tended to report pain and problems with mental well-being as not severe. For example, several reported they were not in pain but did experience an ‘ache’, ‘twinge’ or ‘niggle’ in their hip. Anxiety, depression or low mood were similarly expressed as not severe.

#### Theme 3: pre-existing contextual factors

Well-being during recovery was affected by factors additional to the surgery. Pre-existing health conditions were common, some of which had more impact than the hip surgery and some interviewees were using mobility aids or home adaptations before their surgery.

#### Theme 4: negative impact of limited healthcare

A few interviewees reported early discharge from hospital, attributed to COVID-19, as negatively impacting recovery. Some had either not seen a physiotherapist, had only seen one in hospital, or had to wait a long time for a physiotherapy appointment. A small number only received a physiotherapy booklet. Those who did receive physiotherapy thought it benefited their recovery.

## Discussion

### Principal findings

In this randomized comparison, the SPAIRE approach was not superior to the standard lateral approach in hip hemiarthroplasty for displaced intracapsular fractures, as assessed by the OHS at 120 days postoperation. Analyses of secondary outcomes showed that participants’ mobility and function, and quality of life were similar both in the short term (3 days postoperation) and longer term (120 days postoperation), regardless of whether they received the SPAIRE or lateral surgical approach. Neither surgical approach conferred benefit over the other in terms of length of acute or total hospital stay, return to prefracture place of residence, or survival within 120 days postoperation. Additional analyses indicated that participants receiving the SPAIRE approach experienced less pain in the early postoperative recovery period, compared with those receiving the lateral approach, as measured using the NPRS.

### Strengths and limitations of study

The HemiSPAIRE trial’s strengths are that it is a pragmatic, multicenter RCT studying function and mobility in hip fracture patients undergoing surgery, which was successfully completed within acute hospital settings, despite challenges posed by the COVID-19 pandemic. It had broad inclusion criteria and did not exclude patients based on cognition level, increasing the generalizability of findings. 29% of participants were cognitively impaired and the mean age of participants was 84.6 years, representing a frail group of people, who can often be hard to reach.^25^ Identification of the importance of postoperative function and mobility to hip fracture patients, by patients themselves, influenced the choice of primary outcome measure. Close collaboration with a patient/carer coapplicant and other members of the public from inception to dissemination ensured a patient focus throughout and gave a patient centric view of the implications of the findings. Anticipated follow-up rates for the primary outcome were achieved. Close monitoring and data review by the TSC during the trial enabled adaptation of the planned analyses so that the main analyses used a SACE method, which was chosen specifically to address the issue of potential bias from differential survival between trial arms. An integrated qualitative study provided insight into participants’ experiences. HemiSPAIRE provides an exemplar of an RCT of a surgical intervention in an older population,^26^ which was inspired by hip fracture patients, closely incorporated PPI throughout, and included appropriate adaptations to the design, data collection and analyses in response to both the COVID-19 pandemic and the considerations needed when following up older participants.

Limitations of the trial include the need to change the original primary outcome (DEMMI) due to COVID-19 restrictions. This would have been a more robust method of assessing function at 120 days with minimal ceiling effect. While the OHS is a well-recognized outcome measure in total hip replacement surgery, it is not specifically validated for use with hip fracture patients, although the DEMMI is.^16^ The DEMMI was retained as a secondary outcome at 3 days, collected by research staff at hospital sites. It was also included as a secondary outcome at 120 days, should guidelines concerning COVID-19 allow in-person assessment to resume during the trial. However, this was not possible, and no DEMMI assessments were carried out at 120 days. The study was not powered to detect differences between trial arms in terms of the number of hip-related complications such as infection, nerve palsy, dislocation and periprosthetic fractures. These are rare events, and no definite conclusions can be made based on this study alone. The trial took place during the COVID-19 pandemic, including when restrictions were in place across all participating sites, which affected participants’ experiences of their time in hospital and the postdischarge recovery period, as well as impacting the length of stay measured for all participants. Qualitative findings indicated that access to physiotherapy was limited and may have impacted recovery; this finding resonated strongly with the PPI group who thought physiotherapy was an important aspect of treatment with implications for functional recovery postoperation.

### Comparison with other studies

To our knowledge, this is the first RCT on the use of the SPAIRE modification of the posterior approach in hip hemiarthroplasty patients. Our recent cohort study included the first consecutive 285 hemiarthroplasty patients receiving the SPAIRE approach at our institution, and 567 hemiarthroplasty patients of similar demographic characteristics receiving the lateral approach during the same period.^13^ This cohort study showed little evidence of a difference between surgical approaches in terms of length of stay, discharge destination, or place of residence at 120 days. There were no concerns with regard to postoperative complications. A recent publication by Yoo *et al* 27 reporting clinical results from 307 elderly patients receiving hip hemiarthroplasty surgery involving a similar modification of the posterior approach, preserving the short external rotators, found a single postoperative dislocation.

### Possible implications for clinicians and policy-makers

Surgeons involved in the care of hip fracture patients requiring hemiarthroplasty who routinely use the posterior approach to the hip may find the SPAIRE technique of interest, given the familiarity with the relevant anatomy and potential for reduced pain in the early postoperative period. Current NICE guidelines recommend surgeons consider the lateral approach in hip hemiarthroplasty in favor of the conventional posterior approach. Findings from this and other studies^13 27 28^ show the SPAIRE modification of the posterior approach is safe and equivalent to the standard lateral approach in terms of the outcomes measured. NICE may wish to consider including the SPAIRE modification of the posterior approach as equivalent to the standard lateral approach in its recommendation for surgery.

Research on the use of the SPAIRE modification of the posterior approach in hip hemiarthroplasty patients is ongoing at our institution, including the NIHR Efficacy and Mechanism Evaluation Program funded HIP Surgical Techniques to Enhance Rehabilitation study, which aims to compare the SPAIRE approach to other posterior approaches to the hip, in terms of patient outcomes in total hip arthroplasty. The challenges that the population of England experienced in accessing community rehabilitation during the COVID-19 pandemic are likely to have impacted patient outcome at 120 days postoperation. Findings from our qualitative work reflected this impact of access to physiotherapy on return to function and should be considered in future work.

## Conclusions

The HemiSPAIRE RCT showed that the SPAIRE approach is not superior to the standard lateral approach in hip hemiarthroplasty for displaced intracapsular fractures, as assessed by the OHS at 120 days. Participants’ mobility and function are similar both in the short term (3 days postoperation) and longer term (120 days postoperation) term, regardless of whether they receive the SPAIRE or lateral surgical approach. Neither surgical approach confers benefit over the other in terms of length of acute or total hospital stay, return to prefracture place of residence, survival within 120 days postoperation, or quality of life at 120 days postoperation. Participants receiving the SPAIRE approach may experience less pain in the early postoperative recovery period, compared with those receiving the standard lateral approach. Modification of the posterior approach in hip hemiarthroplasty following the SPAIRE technique can be carried out with equivalent patient outcomes to the lateral approach within 120 days postoperation.

We thank the contribution of patients and trial team members at each recruitment site; members of the trial steering committee (Ashley Blom, Paul Tanner, Rosemary Greenwood); Diana Silcock who provided PPI support during the development and conduct of the HemiSPAIRE trial; and Professor Willie Hamilton and Professor Obioha Ukoumunne who provided overall guidance and feedback on the manuscript.

### Dissemination to participants and related patient and public communities

We have worked closely with the project’s patient advisory group throughout the trial, and this group has helped to shape this paper and the implications of our findings. With this group, we have produced a plain language summary report of the findings (included in online supplemental material) and discussed key messages, who they should be disseminated to and how. We will continue to work with this group to ensure findings reach patients, carers and clinicians. The plain language summary will be published on the PenARC website.

10.1136/bmjsit-2023-000251.supp1 Abstract translation This web only file has been produced by the BMJ Publishing Group from an electronic file supplied by the author(s) and has not been edited for content.

## Data Availability

Data are available on reasonable request. The datasets generated in the HemiSPAIRE randomized controlled trial will be available in the University of Exeter institutional repository, Open Research Exeter (ORE) (https://ore.exeter.ac.uk/repository/). Data will be available within 6 months following publication. Access to the data will be restricted to ensure that data are only made available to bona fide researchers for ethically approved research projects, on the understanding that confidentiality will be maintained and a data access agreement has been signed by an institutional signatory. The lead author affirms that this manuscript is an honest, accurate and transparent account of the study being reported; that no important aspects of the study have been omitted; and that any discrepancies from the study as planned (and, if relevant, registered) have been explained.
