# Supplementary material for: Clinical effectiveness of a modified muscle sparing posterior technique compared with a standard lateral approach in hip hemiarthroplasty for displaced intracapsular fractures (HemiSPAIRE): a multicenter, parallel-group, randomized controlled trial
Source: BMJ Surg Interv Health Technol. 2024 Jun 17;6(1):e000251. doi: 10.1136/bmjsit-2023-000251 (PMC11184196; doi:10.1136/bmjsit-2023-000251)
Supplement: Supplementary data [file bmjsit-2023-000251supp002.pdf]

Supplementary tables

Table S1: Comparison of trial participants and eligible non-participants

Characteristic	Trial participants (N = 244)	Eligible non-participants (N = 702)*
Sex		
Female, n (%)	168 (68.85)	466 (66.38)
Male, n (%)	76 (31.15)	236 (33.62)
Age in years, mean (SD) / range	84.58 (7.18) / 62 to 102	84.43 (7.83) / 60 to 103

SD = standard deviation.

*Eligible non-participants comprise all participants who were screened and excluded prior to randomisation, not explicitly due to not meeting one or more of the inclusion criteria.

Table S2: Baseline characteristics by 120 day survival and primary outcome follow-up status, by trial arm and overall

	**SPAIRE**			**Lateral**			**Overall**		
**Variable**	**Survivors followed up within data collection window N = 90[*]**	**Survivors not followed up within data collection window N = 17[†]**	**Non-survivors N = 15[‡]**	**Survivors followed up within data collection window N = 85[*]**	**Survivors not followed up within data collection window N = 15[†]**	**Non-survivors N = 22[‡]**	**Survivors followed up within data collection window N = 175[*]**	**Survivors not followed up within data collection window N = 32[†]**	**Non-survivors N = 37[‡]**
Gender									
Female, n (%)	64 (71.11)	12 (70.59)	9 (60)	60 (70.59)	7 (46.67)	16 (72.73)	124 (70.86)	19 (59.38)	25 (67.57)
Male, n (%)	26 (28.89)	5 (29.41)	6 (40)	25 (29.41)	8 (53.33)	6 (27.27)	51 (29.14)	13 (40.63)	12 (32.43)
Age in years, mean (SD)	83.67 (7.40)	85.71 (7.16)	87.93 (6.52)	84.51 (7.34)	82.07 (7.97)	87.14 (3.93)	84.07 (7.36)	84.00 (7.65)	87.46 (5.07)
Ethnicity									
White British, n (%)	90 (100)	17 (100)	15 (100)	85 (100)	14 (93.33)	22 (100)	175 (100)	31 (96.88)	37 (100)
American, n (%)					1 (6.67)			1 (3.13)	
Cognition level									
Impaired, n (%)	20 (22.22)	6 (35.29)	10 (66.67)	20 (23.53)	1 (6.67)	14 (63.64)	40 (22.86)	7 (21.88)	24 (64.86)
Not impaired, n (%)	70 (77.78)	11 (64.71)	5 (33.33)	65 (76.47)	14 (93.33)	8 (36.36)	135 (77.14)	25 (78.13)	13 (35.14)
Place of residence									
Own home, n (%)	78 (86.67)	13 (76.47)	7 (46.67)	72 (84.71)	13 (86.67)	11 (50.00)	150 (85.71)	26 (81.25)	18 (48.65)
Residential/supported living[§], n (%)	8 (8.89)	4 (23.53)	6 (40.00)	12 (14.12)	2 (13.33)	9 (40.91)	20 (11.43)	6 (18.75)	15 (40.54)
Nursing home, n (%)	4 (4.44)		2 (13.33)	1 (1.18)		2 (9.09)	5 (2.86)		4 (10.81)
ASA score									
1, n (%)	1 (1.11)						1 (0.57)		
2, n (%)	28 (31.11)	5 (29.41)	1 (6.67)	27 (31.76)		5 (23.81)	55 (31.43)	5 (15.63)	6 (16.67)
3, n (%)	51 (56.67)	11 (64.71)	11 (73.33)	46 (54.12)	14 (93.33)	13 (61.90)	97 (55.43)	25 (78.13)	24 (66.67)
4+, n (%)	10 (11.11)	1 (5.88)	3 (20.00)	12 (14.12)	1 (6.67)	3 (14.29)	22 (12.57)	2 (6.25)	6 (16.67)
OHS, mean (SD)	39.23 (8.05)	39.79 (6.34)	32.91 (4.91)	38.34 (8.09)	36.25 (10.18)	34.47 (5.76)	38.80 (8.06)	38.15 (8.35)	33.86 (5.40)

EQ-5D index value, mean (SD)	0.82 (0.17)	0.74 (0.25)	0.55 (0.25)	0.79 (0.22)	0.67 (0.28)	0.68 (0.23)	0.80 (0.19)	0.71 (0.26)	0.63 (0.24)
EQ-5D VAS, mean (SD)	70.51 (20.23)	58.75 (22.07)	63.57 (19.94)	68.27 (20.87)	59.00 (25.12)	62.5 (26.41)	69.44 (20.50)	58.88 (23.12)	62.89 (23.65)

[*]Defined as those participants who are alive, with data on the primary outcome OHS at 120 days, collected within data collection window of 110 to 130 days.
[†]Defined as those participants who are alive, with missing data on the primary outcome OHS at 120 days, or data on the primary outcome OHS collected outside of the data collection window.
[‡]Defined as those participants who died before 120 day follow-up.
[§]Includes living with family or friends, retirement home/flat/village and community mental health hospital.
Sample sizes for OHS: survivors followed up group: 162 (83 SPAIRE; 79 lateral), survivors not followed up group: 26 (14 SPAIRE; 12 lateral), non-survivors group: 28 (11 SPAIRE; 17 lateral).
Sample sizes for EQ5D-5L index value: survivors followed up group: 148 (76 SPAIRE; 72 lateral), survivors not followed up group: 27 (15 SPAIRE; 12 lateral), non-survivors group: 20 (8 SPAIRE; 12 lateral).
Sample sizes for EQ5D-5L VAS: survivors followed up group: 140 (73 SPAIRE; 67 lateral), survivors not followed up group: 24 (12 SPAIRE; 12 lateral), non-survivors group: 19 (7 SPAIRE; 12 lateral).
SPAIRE = Save Piriformis and Internus, Repairing Externus; SD = standard deviation; ASA = American Society of Anaesthesiologists; OHS = Oxford Hip Score; EQ5D-5L = Five-level EQ-5D; VAS = visual analogue scale.

Table S3: Additional analyses comparing secondary outcomes between SPAIRE and lateral trial arms using survivors analyses

**Outcome**	**SPAIRE**		**Lateral**		**Statistic**	**Unadjusted**	**Adjusted**[§]		
	**N**[*]	**mean (SD) / n (%)**[*]	**N**[*]	**mean (SD) / n (%)**[*]		**estimate**	**estimate**	**95% CI**	**p-value**
Complete case data									
EQ-5D-5L: 120 days									
Index value	83	0.76 (0.24)	82	0.73 (0.22)	Mean difference	0.02	0.02	-0.05 to 0.09	0.52
VAS	77	66.65 (19.65)	74	69.53 (19.60)	Mean difference	-2.88	-3.02	-9.40 to 3.37	0.35
Length of stay (acute)	115	14.05 (16.11)	116	12.66 (7.45)	Mean difference	1.40	1.35	-1.82 to 4.53	0.40
Length of stay (total)	114	19.41 (20.95)	115	19.28 (14.95)	Mean difference	0.13	0.41	-4.20 to 5.02	0.86
DEMMI: 3 days	104	23.25 (13.82)	103	21.96 (14.99)	Mean difference	1.29	1.19	-2.16 to 4.54	0.48
CAS: 3 days	110	6.64 (3.69)	108	6.50 (3.60)	Mean difference	0.14	0.03	-0.81 to 0.87	0.95
Discharged from acute stay to pre-fracture residence	115	73 (63.48)	116	63 (54.31)	Odds ratio	1.46	1.50	0.84 to 2.68	0.17
Living in same place of residence at 120 days as pre-fracture	90	76 (84.44)	87	73 (83.91)	Odds ratio	1.04	1.07	0.46 to 2.47	0.88
	**N**[†]		**N**[†]						
Multiply imputed data sets									
EQ-5D-5L: 120 days									
Index value	98		94		Mean difference	0.01	0.01	-0.06 to 0.08	0.70
VAS	98		95		Mean difference	-2.95	-2.90	-9.10 to 3.30	0.36
Length of stay (acute)	116		117		Mean difference	1.39	1.49	-2.56 to 5.54	0.47
Length of stay (total)	115		116		Mean difference	0.14	0.55	-4.70 to 5.80	0.84
DEMMI: 3 days	115		115		Mean difference	1.27	0.80	-2.45 to 4.06	0.63
CAS: 3 days	114		113		Mean difference	0.15	0.06	-0.78 to 0.91	0.88
Discharged from acute stay to pre-fracture residence	116		117		Odds ratio	1.47	1.51	0.84 to 2.71	0.17
Living in same place of residence at 120 days as pre-fracture	92		92		Odds ratio	1.05	1.08	0.47 to 2.50	0.85

SPAIRE = Save Piriformis and Internus, Repairing Externus; SD = standard deviation; CI = confidence interval; EQ-5D-5L = Five-level EQ-5D; VAS = visual analogue scale; DEMMI = de Morton mobility index; CAS = cumulated ambulatory score.

[*]Sample sizes and summary statistics from original trial data.

[†]Sample sizes after multiple imputation.

[§]For EQ-5D-5L, lengths of stay, DEMMI, CAS and discharged from acute stay to pre-fracture place of residence: adjusted for site, cognition level and pre-fracture characteristics age, gender, place of residence, ASA score. For living in same place of residence at 120 days as pre-fracture: adjusted for site and cognition level.

Table S4: Additional analyses comparing secondary outcomes between SPAIRE and lateral trial arms using the composite approach

Outcome	SPAIRE		Lateral		Statistic	Unadjusted	Adjusted[§]		
	N[*]	mean (SD) / n (%)[*]	N[*]	mean (SD) / n (%)[*]		estimate	estimate	95% CI	p-value
Complete case data									
EQ-5D-5L: 120 days									
Index value	98	0.64 (0.35)	104	0.58 (0.36)	Mean difference	0.06	0.04	-0.05 to 0.13	0.38
VAS	92	55.78 (30.58)	96	53.59 (34.03)	Mean difference	2.19	-0.60	-9.09 to 7.89	0.89
Length of stay (acute)	121	19.16 (27.39)	121	16.97 (22.09)	Mean difference	2.19	3.01	-3.03 to 9.05	0.33
Length of stay (total)	121	25.81 (32.95)	121	24.77 (28.20)	Mean difference	1.04	2.11	-5.27 to 9.49	0.57
DEMMI: 3 days	106	22.81 (14.05)	105	21.54 (15.15)	Mean difference	1.27	1.13	-2.17 to 4.44	0.50
CAS: 3 days	112	6.52 (3.76)	110	6.38 (3.67)	Mean difference	0.14	0.02	-0.81 to 0.84	0.97
Discharged from acute stay to pre-fracture residence	121	73 (60.33)	121	63 (52.07)	Odds ratio	1.40	1.42	0.81 to 2.49	0.23
Living in same place of residence at 120 days as pre-fracture	105	76 (72.38)	109	73 (66.97)	Odds ratio	1.29	1.30	0.69 to 2.46	0.42
	N[†]		N[†]						
Multiply imputed data sets									
EQ-5D-5L: 120 days									
Index value	105		109		Mean difference	0.06	0.04	-0.04 to 0.13	0.34
VAS	105		109		Mean difference	1.73	0.21	-7.69 to 8.11	0.96
Length of stay (acute)	122		122		Mean difference	2.64	2.81	-2.86 to 8.49	0.33
Length of stay (total)	122		122		Mean difference	2.15	2.35	-3.97 to 8.67	0.47
DEMMI: 3 days	112		112		Mean difference	1.75	1.04	-2.26 to 4.33	0.54
CAS: 3 days	112		112		Mean difference	0.25	0.03	-0.82 to 0.88	0.94
Discharged from acute stay to pre-fracture residence	122		122		Odds ratio	1.33	1.35	0.76 to 2.41	0.31
Living in same place of residence at 120 days as pre-fracture	105		109		Odds ratio	1.44	1.34	0.69 to 2.63	0.39

SPAIRE = Save Piriformis and Internus, Repairing Externus; SD = standard deviation; CI = confidence interval; EQ-5D-5L = Five-level EQ-5D; VAS = visual analogue scale; DEMMI = de Morton mobility index; CAS = cumulated ambulatory score.
[*]Sample sizes and summary statistics from original trial data.
[†]Sample sizes after multiple imputation.

[§]For EQ-5D-5L, lengths of stay, DEMMI, CAS and discharged from acute stay to pre-fracture place of residence: adjusted for site, cognition level and pre-fracture characteristics age, gender, place of residence, ASA score. For living in same place of residence at 120 days as pre-fracture: adjusted for site and cognition level.

Table S5: Additional analyses comparing secondary outcomes between SPAIRE and lateral trial arms using the survivor average causal effect approach

Outcome	SPAIRE		Lateral		Statistic	Unadjusted	Adjusted§		
	N*	mean (SD) / n (%)*	N*	mean (SD) / n (%)*		estimate	estimate	95% CI	p-value
Complete case data									
Including data collected outside data collection window									
EQ-5D-5L: 120 days									
Index value	92	0.75 (0.24)	88	0.73 (0.22)	Mean difference	0.02	0.02	-0.05 to 0.08	0.58
VAS	86	65.55 (20.66)	79	68.99 (19.45)	Mean difference	-3.20	-3.42	-9.56 to 2.72	0.27
Living in same place of residence at 120 days as pre-fracture	105	84 (80.00)	95	79 (83.16)	Odds ratio	0.88	0.90	0.42 to 1.93	0.78
Excluding data collected by proxy									
EQ-5D-5L: 120 days									
Index value	45	0.75 (0.26)	35	0.75 (0.17)	Mean difference	-0.01	-0.01	-0.11 to 0.09	0.85
VAS	43	69.60 (18.56)	33	69.48 (19.72)	Mean difference	-0.01	-0.55	-9.05 to 7.95	0.90
	N†		N†						
Multiply imputed data sets									
EQ-5D-5L: 120 days									
Index value	98		94		Mean difference	0.01	0.01	-0.05 to 0.08	0.71
VAS	98		95		Mean difference	-3.11	-2.85	-9.01 to 3.32	0.36
Length of stay (acute)	116		117		Mean difference	1.77	1.68	-2.52 to 5.89	0.43
Length of stay (total)	115		116		Mean difference	1.22	1.25	-4.29 to 6.79	0.66
Discharged from acute stay to pre-fracture residence	116		117		Odds ratio	1.40	1.43	0.79 to 2.61	0.24
Living in same place of residence at 120 days as pre-fracture	92		92		Odds ratio	1.14	1.14	0.47 to 2.75	0.77

SPAIRE = Save Piriformis and Internus, Repairing Externus; SD = standard deviation; CI = confidence interval; EQ-5D-5L = Five-level EQ-5D; VAS = visual analogue scale; DEMMI = de Morton mobility index; CAS = cumulated ambulatory score.

*Sample sizes and summary statistics from original trial data.

†Sample sizes after multiple imputation.

§For EQ-5D-5L, lengths of stay, DEMMI, CAS and discharged from acute stay to pre-fracture place of residence: adjusted for site, cognition level and pre-fracture characteristics age, gender, place of residence, ASA score. For living in same place of residence at 120 days as pre-fracture: adjusted for site and cognition level.
