# Supplementary material for: Clinical effectiveness of a modified muscle sparing posterior technique compared with a standard lateral approach in hip hemiarthroplasty for displaced intracapsular fractures (HemiSPAIRE): a multicenter, parallel-group, randomized controlled trial
Source: BMJ Surg Interv Health Technol. 2024 Jun 17;6(1):e000251. doi: 10.1136/bmjsit-2023-000251 (PMC11184196; doi:10.1136/bmjsit-2023-000251)
Supplement: Abstract translation [file bmjsit-2023-000251supp001.pdf]

Plain language summary

Which approach is best for partial hip replacement?

Background: When someone breaks their hip, they often need a partial hip replacement. To perform this operation surgeons, need to cut through some muscles to expose the hip joint. Current guidelines suggest that the surgeon cuts muscles and tendons from the side of the hip during the operation (Standard approach). Alternatively, you can use a modified technique that leaves all the major muscles intact (SPAIRE approach). There is no research evidence to show which approach is better.

Aim of the study: To compare two ways of performing partial hip replacement to see which one allows patients to move with better function after surgery.

Study design: Patients who agreed to be part of the study were randomly allocated to have one of the two techniques for their partial hip replacement. We compared the impact of the two techniques on how quickly and fully patients mobilised, the level of pain they experienced, and if there were any complications after surgery. We talked to patients from both groups to hear about their recovery experience.

Participants: 244 adults (aged ≥ 60 years) who required a partial hip replacement after hip fracture. 122 participants received the standard approach, and 122 received the modified SPAIRE approach.

Findings: The participants' mobility and function were the same for both surgical approaches. This was true in the short term, 3 days after surgery, and in the longer term, 120 days after surgery. The two approaches were similar for length of hospital stay, return home, survival, and quality of life. Participants receiving the modified (SPAIRE) approach may experience less pain in the early (3 days post-surgery) recovery period. This is compared to those receiving the lateral approach. Participants in both groups gave similar accounts of experience of recovery after surgery. Participants described gradual improvements in return to normal functions and emphasised the importance of physiotherapy in the recovery process.

Conclusions: Both approaches to partial hip replacement offer similar outcomes in terms of mobility and function.
